# Population-Level Impact of Same-Day Microscopy and Xpert MTB/RIF for Tuberculosis Diagnosis in Africa

**DOI:** 10.1371/journal.pone.0070485

**Published:** 2013-08-12

**Authors:** David W. Dowdy, J. Lucian Davis, Saskia den Boon, Nicholas D. Walter, Achilles Katamba, Adithya Cattamanchi

**Affiliations:** 1 Department of Epidemiology, Johns Hopkins Bloomberg School of Public Health, Baltimore, Maryland, United States of America; 2 Division of Pulmonary and Critical Care Medicine, San Francisco General Hospital, University of California San Francisco, San Francisco, California, United States of America; 3 Curry International Tuberculosis Center, San Francisco General Hospital, University of California San Francisco, San Francisco, California, United States of America; 4 Makerere University-University of California San Francisco Research Collaboration, Kampala, Uganda; 5 Division of Pulmonary and Critical Care Medicine, University of Colorado Denver, Aurora, Colorado, United States of America; 6 Department of Medicine, Makerere University, Kampala, Uganda; University of Cape Town, South Africa

## Abstract

**Objective:**

To compare the population-level impact of two World Health Organization-endorsed strategies for improving the diagnosis of tuberculosis (TB): same-day microscopy and Xpert MTB/RIF (Cepheid, USA).

**Methods:**

We created a compartmental transmission model of TB in a representative African community, fit to the regional incidence and mortality of TB and HIV. We compared the population-level reduction in TB burden over ten years achievable with implementation over two years of same-day microscopy, Xpert MTB/RIF testing, and the combination of both approaches.

**Findings:**

Same-day microscopy averted an estimated 11.0% of TB incidence over ten years (95% uncertainty range, UR: 3.3%–22.5%), and prevented 11.8% of all TB deaths (95% UR: 7.7%–27.1%). Scaling up Xpert MTB/RIF to all centralized laboratories to achieve 75% population coverage had similar impact on incidence (9.3% reduction, 95% UR: 1.9%–21.5%) and greater effect on mortality (23.8% reduction, 95% UR: 8.6%–33.4%). Combining the two strategies (i.e., same-day microscopy plus Xpert MTB/RIF) generated synergistic effects: an 18.7% reduction in incidence (95% UR: 5.6%–39.2%) and 33.1% reduction in TB mortality (95% UR: 18.1%–50.2%). By the end of year ten, combining same-day microscopy and Xpert MTB/RIF could reduce annual TB mortality by 44% relative to the current standard of care.

**Conclusion:**

Scaling up novel diagnostic tests for TB and optimizing existing ones are complementary strategies that, when combined, may have substantial impact on TB epidemics in Africa.

## Introduction

Tuberculosis (TB) remains a leading infectious cause of death worldwide, contributing to over 1.4 million deaths annually; yet 35% of all cases go undetected, and an additional 7% are diagnosed too late to prevent death [1]. The burden of TB is most profound in sub-Saharan Africa (World Health Organization [WHO] African Region), where incidence rates are over twice the global average [1], and the Millennium Development Goals' 2015 target of a 50% reduction in TB prevalence and mortality from 1990 rates is unlikely to be reached [2], [3].

A key contributor to this burden of morbidity and mortality is poor diagnosis; only 60% of new TB cases in Africa are ever detected [1]. Inadequate case detection reflects, in part, the limitations of sputum smear microscopy, the primary test used for TB diagnosis in high-burden countries. Specifically, sputum smear microscopy, as currently implemented, has two key shortcomings. First, its sensitivity is limited [4], [5], missing about half of all cases. Second, many people with smear-positive TB do not initiate treatment because they cannot complete the standard multi-day process of sputum collection, testing, and reporting results.

Since 2010, the WHO has issued policy guidelines to mitigate both of these weaknesses. To reduce losses to follow-up, the WHO endorsed “same-day microscopy,” recommending that systems be developed to collect sputum, perform microscopy, report results, and initiate treatment on the day of initial presentation [6]. To improve the sensitivity of diagnosis, the WHO endorsed the Xpert MTB/RIF test (“Xpert”, Cepheid, Inc., Sunnyvale, California, USA), an automated molecular assay with minimal human-resource requirements and the ability to detect 70% of TB that is negative by sputum smear [7]. Although Xpert can be performed in 90 minutes, it is currently too expensive, and has too many technical requirements (e.g., stable electrical supply) to deploy at the most peripheral level in most African settings [8]. As such, “same-day Xpert” is a technically feasible strategy, but one that requires additional infrastructure (e.g., rapid sputum transport and reporting of results) and thus is unlikely to be immediately implementable in most African settings. Since both same-day diagnosis and scale-up of Xpert entail substantial financial and logistical burdens, public health officials in high-burden countries are now faced with the decision of which recommendation to prioritize.

In making this decision, one important consideration for policymakers is the projected impact of each approach on TB incidence and mortality. Epidemiological models can provide valuable insight in this regard [9]. We therefore constructed a mathematical model of the TB epidemic among adults in a setting representative of the WHO African Region, in order to compare the population-level impact achievable by scale-up of Xpert, implementation of same-day microscopy, and both strategies combined.

## Methods

**Figure 1:**
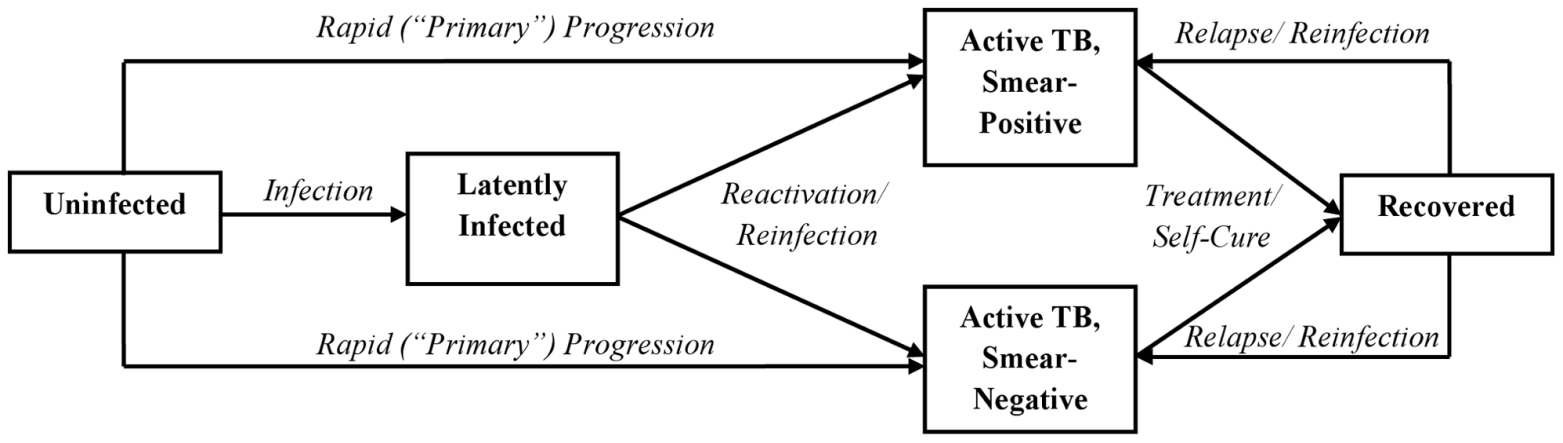
Model Compartmental Structure. Upon infection with TB, susceptible individuals may progress rapidly to active TB or enter a state of latent infection, from which active TB can develop at any time. Reinfection of latently-infected individuals occurs, as do relapse and reinfection after treatment/recovery. Smear-negative disease is modeled as a weighted average of smear-negative pulmonary and extrapulmonary disease. The model population is also subdivided by HIV status (positive vs. negative); people living with HIV are at increased risk of primary progression and reactivation of latent TB, are more likely to have smear-negative disease, and experience higher mortality rates (both TB and non-TB mortality).

We constructed a compartmental model of a generalizable African TB epidemic using ordinary differential equations, with a structure (Figure 1) based on that of other published models of TB [10], [11]. Our goal was to generate an accessible, reproducible model that relies on a minimum of parameter assumptions to estimate the TB epidemiological burden potentially avertable by implementation of TB diagnostic interventions in the WHO African Region. Thus, rather than modeling precise implementation in one specific setting, our model aims to provide policymakers in many settings a general framework for considering the tradeoffs between Xpert and same-day microscopy. Although we provide results for a generic setting that is representative of the WHO African Region, we also provide model code that can be modified to fit local epidemics in most HIV-endemic settings.

Individuals enter the model at age 15 and exit at death. Upon infection, individuals progress either to latent infection or active TB, which is modeled as a combination of smear-positive (i.e., could be detected using sputum smear alone in an idealized laboratory environment) and smear-negative (modeled as a weighted average of smear-negative pulmonary and extrapulmonary) disease. Individuals with active TB undergo diagnostic attempts at a constant rate, and the success of each attempt depends on the diagnostic sensitivity (conditional on smear status), probability of loss to follow-up before treatment initiation, and probability of treatment success after initiation. Successful diagnostic attempts (i.e., those that ultimately lead to treatment completion or cure) result in immediate reduction of mortality risk and elimination of infectiousness. Unsuccessful attempts result in return to the active infectious pool. Reactivation of latent disease, reinfection, and relapse all occur at defined rates. Information S1, S2, and S3 provide the model equations/description, code, and equilibrium population, respectively.

We incorporated HIV status (positive versus negative), assuming that people living with HIV have a higher probability of developing active TB (from initial infection and reactivation) and undergo diagnostic attempts more often, as the symptoms of their disease are more severe. However, people living with HIV are also more likely to have smear-negative TB and are more likely to die, either from TB or from other causes. Because the logistics of diagnosing and treating drug-resistant TB are complex (and not undertaken to a great extent in Africa), and data on rates of drug resistance outside of South Africa are sparse, we did not explicitly include drug resistance in this analysis. However, since the number of drug resistant cases detected by Xpert may be a key consideration in decision-making, we do provide estimates of this quantity as a function of the overall prevalence of drug resistance among incident TB cases over the subsequent ten years in any given local setting.

We fit the model to a generic population representative of the WHO African Region by bringing the population to equilibrium in 2002, using an iterative routine [12] to create a population with identical values to WHO estimates for six epidemiological data points in that year: population size, TB incidence, TB incidence among people living with HIV, TB mortality, HIV-associated TB mortality, and HIV prevalence. Each epidemiological data point was matched to a single model parameter in one-to-one fashion as described in Table 1. We then assumed a linear change in each parameter value such that the model again replicated WHO estimates for each data point in 2010, the last year for which data were available. In the reference scenario, these parameters were assumed to continue their same linear trajectories through 2022, except for population growth, which we assumed (for simplicity) to remain constant at 2.25% per year after 2010. We then modeled the following four scenarios:

**Table 1:** Fitting of Dynamic Model Parameters to Epidemiological Data.

Epidemiological Data Point	Value, Africa^a^	Value, Model	Corresponding Model Parameter	Parameter Value	Range^b^
Population size, millions	2002: 401	2002: 401	Percentage increase in population, per year	2002: 2.32%	1.5–3.0%
	2010: 485	2010: 485		2010: 2.25%	
		2022: 638		2022: 2.25%	
TB incidence, per 100,000/year	2002: 515	2002: 515	Number of transmissions per highly-infectious TB case, per year	2002: 11.1	10.0–12.0
	2010: 445	2010: 445		2010: 10.9	
		2022: 333		2022: 10.7	
HIV/TB incidence, per 100,000/year	2002: 247	2002: 247	Relative risk of infection, HIV positive^c^	2002: 3.67	1.0–10.0
	2010: 183	2010: 183		2010: 2.46	
		2022: 89		2022: 1.0^c^	
HIV prevalence	2002: 5.03%	2002: 5.03%	HIV incidence rate, per 1,000/yr	2002: 3.3	1.6–6.6
	2010: 4.08%	2010: 4.08%		2010: 2.5	
		2022: 2.84%		2022: 1.4	
TB mortality, per 100,000/year	2002: 56	2002: 56	Rate of diagnostic attempts, HIV-negative, per year	2002: 0.80	0.4–1.6
	2010: 48	2010: 48		2010: 1.15	
		2022: 33		2022: 1.66	
HIV/TB mortality, per 100,000/year	2002: 79	2002: 79	Rate of diagnostic attempts, HIV- positive, per year	2002: 18.1	4.0–25.0
	2010: 55	2010: 55		2010: 17.2	
		2022: 27		2022: 15.9	

a Adjusted to reflect the adult (rather than total) population of the WHO African Region. Values were adjusted from reference [13], except for population size [14] and HIV prevalence [15].

b Sensitivity range applies to the 2002 “intercept” value; “slopes” from 2002 to 2022 were varied by a factor of two in either direction for multivariable uncertainty analysis but were not included in the one-way sensitivity analysis (as they are difficult to compare to other parameters).

c Accounts for the fact that HIV-infected individuals have higher risk of disease due to recent infection than strictly predicted from theoretical studies (e.g., due to residence in geographic “hotspots,” risk of nosocomial infection, household clustering, higher rates of smoking and other TB determinants, etc.). Values lower than 1.0 were not allowed, as they are implausible.

- Standard of Care: 15% of people with TB presenting for evaluation are lost to follow-up before initiating treatment (based on the median value from a literature review [16]–[24]);
- Same-day Microscopy: Optimization of sputum smear to provide results on the same day as initial presentation (thereby reducing the proportion of people with smear-positive TB who are lost to follow-up before starting treatment [so-called “initial default”] from 15% to 1.5%), scaled-up over two years, starting in 2013;
- Xpert MTB/RIF: Replacement of sputum smear with Xpert (resulting in increased sensitivity for smear-negative TB from 0% to 72% [25]) over two years, starting in 2013, and achieving a maximum coverage of 75% of the population; and
- Same-day Microscopy plus Xpert: Simultaneous implementation of same-day diagnosis (modeled as a reduction in pre-treatment loss to follow-up for smear-positive TB) and scale-up of Xpert MTB/RIF as a replacement for smear microscopy (modeled as an increase in sensitivity for smear-negative TB) over two years, starting in 2013.

Under each scenario, we assume that a similar proportion of individuals who test negative with the primary test (sputum smear microscopy or Xpert) will nonetheless be diagnosed with TB and started on therapy, due to a combination of high clinical suspicion and results of ancillary testing (e.g., chest X-ray). This proportion of people with TB who are treated empirically is assumed to remain constant regardless of whether Xpert or smear is used for primary diagnosis.

Additional model parameters were estimated from the literature and derived from WHO estimates for the African Region (Table 2). Our primary outcomes were ten-year TB incidence and mortality, stratified by HIV status. We performed one-way sensitivity analyses on all model parameters by varying each parameter over a reasonable range, based on existing knowledge of TB (Tables 1 and 2). We also performed probabilistic uncertainty analyses by simultaneously (using Latin Hypercube sampling) varying all parameters over a beta distribution with alpha  = 4, upper and lower bounds defined as in Tables 1 and 2, and the most likely value as the mode. We report 95% uncertainty ranges as the 2.5^th^ and 97.5^th^ percentiles of results from 1000 simulations.

**Table 2:** Additional Static Model Parameters.

Parameter	Value	Sensitivity Range	Reference
Proportion of infections progressing rapidly:^a^			
HIV-negative	0.14	0.08–0.2	[26]
HIV-positive	0.4	0.2–1.0	[27], [28]
Reduction in proportion of rapid progression among HIV-negatives with latent infection	0.5	0.3–0.7	[26], [29]
Reactivation rate after latent infection, per year:			
HIV-negative	0.00058	0.0001–0.001	[30]
HIV-positive	0.0331	0.01–0.1	[30]
Proportion of TB that is smear-positive:			
HIV-negative^b^	0.65	0.5–0.75	[4], [5], [13]
HIV-positive^c^	0.5	0.3–0.55	[31]
Relative infectiousness of smear-negative TB^d^	0.15	0–0.23	[13], [32]
Proportion of smear-positive cases missed (i.e., false negative) with sputum microscopy	0.05	0–0.15	[33]
Proportion of TB cases lost to follow-up before treatment initiation (“initial default”)	0.15	0.1–0.2	[16]–[24]
Proportion of cases treated empirically^e^	0.25	0–0.5	[13]
Treatment success proportion	0.8	0.7–0.85	[13]
Rate of spontaneous recovery, per year:^f^			
Smear-negative	0.264	0.1–0.4	[34]
Smear-positive	0.099	0.05–0.2	[34]
Mortality rate, per year:			
HIV-positive, no TB	0.053	0.025–0.1	[35]
HIV-negative, smear-positive TB	0.231	0.15–0.4	[34]
HIV-negative, smear-negative TB	0.066	0.034–0.115	[34]
HIV-positive, any form of TB	2.0	1.0–4.0	[36]
Life expectancy at age 15, in years	46.5	30–60	[37]

a “Rapid” progression incorporates all active TB that develops within five years after infection.

b Taken as the median sensitivity of “optimized” microscopy (i.e., concentrated sputum or fluorescent microscopy) for pulmonary disease, reduced by the 5% of TB in HIV-uninfected individuals that is extrapulmonary (estimated using notifications to the WHO Western Pacific Region, where HIV prevalence is low).

c Taken as the median of studies from HIV-infected individuals in the cited review.

d Assumes that smear-negative pulmonary TB is 0.22 times as infectious as smear-positive pulmonary TB [32] and that one-third of smear-negative TB is extrapulmonary [13].

e Fit at baseline to provide the proportion of notified new TB cases in the WHO African Region that are smear-positive (0.56).

f Assumed to be zero if HIV-infected.

## Results

### Standard of Care Scenario

#### TB incidence

**Figure 2:**
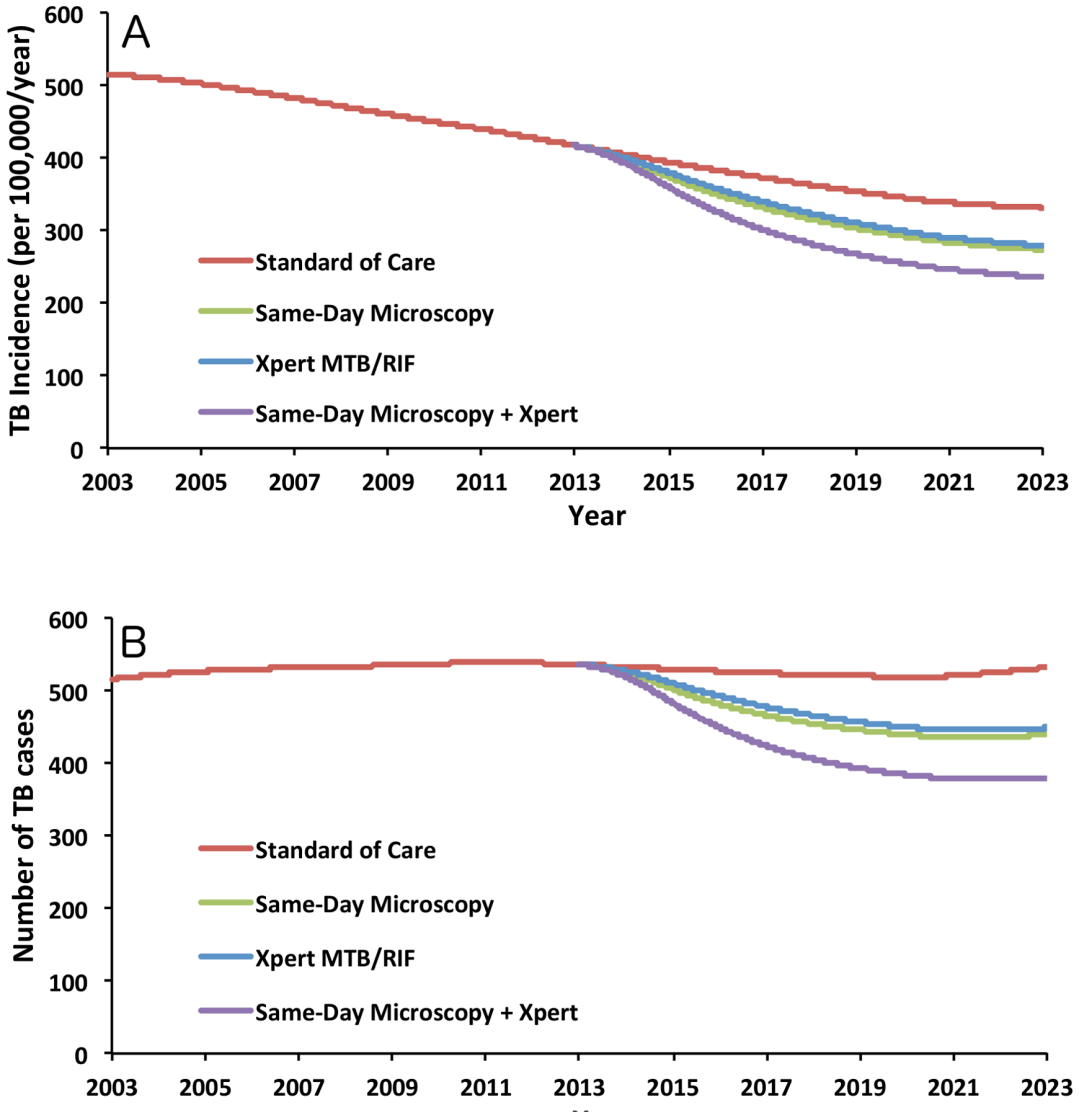
Projected Trajectory of TB Incidence in Africa, 2013–2022. Panel A shows the TB incidence rate (per 100,000 population per year), while Panel B shows the projected number of TB cases per year in an area with an adult population of 10 million in 2002, assuming constant 2.25% population growth.

Over the ten-year period from 2013 to 2022, adult TB incidence was projected to fall from 417 to 331 per 100,000/year in the absence of additional diagnostic interventions (Figure 2A). This decline in incidence was evenly matched by an estimated 2.25% annual increase in population size, such that the annual number of estimated TB cases remained stable throughout the projected time period, increasing by 3.2% from 2003 to 2022 (Figure 2B).

#### Mortality

The adult TB mortality rate fell from 92.4 to 59.2 per 100,000/year. Because we assumed a continued linear decline in HIV incidence, HIV-associated TB accounted for a decreasing proportion of TB deaths, from 52% in 2013 to 44% in 2022. By contrast, the annual number of TB deaths in adults without HIV remained relatively stable, declining by only 5.4% from 2003 to 2022.

### Same-Day Microscopy Scenario

#### TB incidence

Implementation of same-day microscopy in 2013–2014 reduced cumulative TB incidence over the ten-year period by 11.0% (95% uncertainty range, UR: 3.3%–22.5%). This reduction in incidence corresponds to 58,000 cases averted in an area with a 2003 adult population of 10 million (Table 3) and intensified over time (Figure 2). By the end of 2022, adult TB incidence had fallen from 331 to 273 per 100,000 per year: a 17.5% reduction (95% UR: 4.2%–33.8%) relative to the standard of care.

**Table 3:** Cumulative Ten-Year Projected Burden of TB (2013–2022) in an African Area with a 2003 Population of 10 Million Adults.

Scenario	Cumulative Incidence		Cumulative Mortality	
	Number of Incident Cases	Percent Reduction (95% UR)	Number of Deaths^a^	Percent Reduction (95% UR)
Existing Standard	525,000	0 (ref)	105,000	0 (ref)
Same-Day Microscopy	467,000	11.0%	92,000	11.8%
		(3.3%–22.5%)		(7.7%–27.1%)
Xpert MTB/RIF (75% coverage)	476,000	9.3%	80,000	23.8%
		(1.9%–21.5%)		(8.6%–33.4%)
Same-Day Microscopy plus Xpert	427,000	18.7%	70,000	33.1%
		(5.6%–39.2%)		(18.1%–50.2%)

UR, uncertainty range.

a Includes TB deaths among people living with HIV.

#### Mortality

Mortality declined by a similar proportion: an 11.8% cumulative reduction (95% UR: 7.7%–27.1%), corresponding to 12,000 lives saved. This mortality reduction was differential according to HIV status, with same-day microscopy averting 16.2% of all TB deaths among HIV-uninfected adults, but only 6.9% of HIV-associated TB deaths.

### Xpert MTB/RIF Scenario

#### TB incidence

Over the ten-year analysis period, scale-up of Xpert MTB/RIF as a first-line diagnostic test – achieving 75% population coverage by January 1, 2015 – averted a similar proportion of TB incidence (9.3% reduction, 95% UR: 1.9%–21.5%) as immediate implementation of same-day microscopy (Figure 2). This impact corresponded to 49,000 cases averted in an area with a 2003 population of 10 million, and a reduction in incidence to 279 per 100,000 per year (15.6% reduction, 95% UR: 2.1%–35.4%) by the end of 2022.

#### Mortality

Scale-up of Xpert reduced cumulative mortality by 23.8% (95% UR: 8.6%–33.4%), a substantially greater impact than seen with same-day microscopy. The mortality benefit of Xpert relative to same-day microscopy was confined to people living with HIV: Xpert averted 17.3% of TB deaths among HIV-uninfected adults versus 16.2% for same-day microscopy, but 30.9% of HIV-associated TB deaths versus 6.9% for same-day microscopy.

#### Resource Requirement

Scale-up of Xpert in an area with a 2003 population of 10 million adults resulted in 325,000 true-positive diagnoses of TB being made by Xpert over the ten-year period from 2013–2022. Thus, if one in ten Xpert test results were positive for TB, 325,000 tests would need to be performed per year on average: assuming a mean volume of 12.5 tests per day and 260 testing days per year from a four-module Xpert machine (i.e., 3,250 tests per machine-year), 100 machines would be required to achieve this level of scale-up. If 2.9% of TB cases were multidrug-resistant (MDR) [1] and Xpert had 94% sensitivity plus 98% specificity for rifampin resistance [7], Xpert would identify 8,860 true-positive and 6,310 false-positive MDR-TB cases over the ten-year span.

### Same-Day Microscopy plus Xpert Scenario

#### TB incidence

A combined diagnostic strategy in which smear-positive patients were treated on the same day, while smear-negative patients had access to centralized Xpert, yielded additive effects on both incidence and mortality, averting 99,000 cases in an area with a 2003 population of 10 million (18.7% of TB incidence, 95% UR: 5.6%–39.2%) and saving 35,000 lives (33.1% of TB mortality, 95% UR: 18.1%–50.2%) (Table 3). Under this strategy, by the end of 2022, TB incidence had fallen to 235 per 100,000/year, a 28.8% reduction (95% UR: 6.2%–60.0%) compared to the standard of care.

#### Mortality

The effect on mortality was even greater, with the combined diagnostic intervention achieving a 44.2% reduction (95% UR: 26.8%–69.5%) in the annual TB mortality rate relative to the standard of care by the end of 2022.

### Sensitivity Analysis

**Figure 3:**
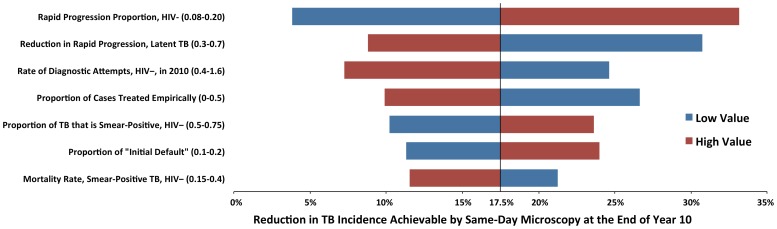
One-Way Sensitivity Analyses. Blue bars represent the low end of the sensitivity range of each parameter, as shown in Tables 1 and 2, and red bars represent the corresponding high values. The outcome was the percentage reduction in TB incidence, comparing the same-day microscopy strategy to the standard of care at the end of 2022; this was estimated at 17.5% in the base case (vertical line in this Figure, corresponds to the difference between green and red lines at the far right of Figure 1). Although sensitivity analysis was performed on all parameters, only those that caused a +/−5% change in the projected value of the outcome are shown here.

The impact of same-day microscopy on TB incidence after ten years was most sensitive to parameters that determined the relative amount of incident TB due to recent infection (versus reactivation of latent disease) and the proportion of TB that would be diagnosed empirically. Same-day microscopy had greater effects in scenarios where more incident TB cases were caused by recent infection and fewer cases were diagnosed empirically (Figure 3). The projected reduction in incidence correlated closely with the proportion of TB cases lost to follow-up after the day of initial presentation (i.e., those benefiting from the same-day strategy); for every 1% of TB cases lost to follow-up in this fashion, same-day microscopy and treatment initiation reduced TB incidence at ten years by an estimated 1.2%.

## Discussion

This mathematical model demonstrates that optimization of an existing tool (sputum smear microscopy) and scale-up of a novel tool (Xpert MTB/RIF) have similar projected population-level impact on TB incidence in communities representative of the WHO African Region. Both same-day microscopy and Xpert, implemented alone, averted 9–11% of TB cases over ten years in our model. Such reductions are likely to vary by epidemiological setting; this simplified, generic model can serve as a starting point for decision-makers across a variety of settings to develop projections that are locally relevant. Although Xpert and same-day microscopy can independently provide important impact, transforming the trajectory of the TB epidemic in Africa will require a combined diagnostic strategy that includes both rapid initiation of treatment for smear-positive patients and improved sensitivity for smear-negative TB (19% modeled reduction in cumulative incidence), in conjunction with other measures for TB prevention, treatment, and risk-factor management.

Our projections of the independent effects of same-day microscopy and of Xpert MTB/RIF are comparable to those of other published models, as well as common-sense calculations. Assuming that 80% of incident TB in Africa is due to recent infection [12], 15% of people with incident TB are lost to follow-up through “initial default,” and smear-negative TB (mostly detectable by Xpert over repeated rounds of diagnosis) accounts for about 15% of all transmission (Table 1), one might expect same-day microscopy and Xpert MTB/RIF to each reduce TB transmission by about 0.8 * 0.15 = 12%, an impact on incidence similar to that actually suggested by the model. Abu-Raddad and colleagues estimated that a novel molecular test could reduce TB incidence by 23% and mortality by 24% in Southeast Asia [38]; in a more directly comparable analysis, Menzies et al estimated that Xpert could reduce 10-year TB incidence by 5% and mortality by 15% in southern Africa [39]. Our estimates of Xpert's impact (reductions of 9% in incidence and 24% in mortality) are somewhat more optimistic but within the corresponding uncertainty ranges, using a model with 10 population compartments of TB and HIV status, rather than over 500 compartments as used by Menzies and colleagues.

This study is among the first to evaluate alternative diagnostic tests for TB using a comparative-effectiveness framework. In the current setting of constrained resources for TB control, studies of comparative effectiveness are increasingly important to decision-makers who must frequently choose between alternative interventions, both of which are globally recommended and likely to be highly cost-effective. We show here that Xpert and same-day microscopy are similarly effective, and complementary, strategies. Combining same-day reporting with scale-up of Xpert MTB/RIF increased population-level impact substantially over either strategy alone and was the only approach that led to substantial gains toward TB elimination during our ten-year analysis period. Similarly, Theron and colleagues compared Xpert alone against Xpert with a series of adjunctive tests, finding that a combination of Xpert and smear microscopy had higher accuracy and lower cost than either test alone [40]. Since the combination of both strategies is substantially more effective than either strategy alone, scale-up of novel diagnostics such as Xpert should not divert attention from optimization of smear microscopy, nor vice versa. However, where resources do not allow implementation of this combined strategy, the choice of which component to prioritize first should depend largely on budget and feasibility considerations, as the population-level effectiveness of the two strategies in isolation is similar.

Optimizing existing diagnostic tests and introducing novel ones represent synergistic strategies for enhancing TB control. Same-day microscopy is a more immediately deployable intervention that can lower TB incidence by reducing losses to follow-up prior to treatment initiation and targeting those individuals who contribute most to TB transmission. Although Xpert is likely to require more resources for scale-up, it has greater benefit for those at highest risk of death, supporting the current recommendation for use of Xpert as a first-line test in people living with HIV [41].

By assuming complete scale-up over two years and very high levels of population coverage (100% for same-day microscopy, 75% for Xpert), this analysis represents an idealized “best-case scenario” and overstates the gains achievable by these interventions in practice. In reality, both ensuring same-day treatment initiation for smear-positive cases and scale-up of Xpert are challenging tasks. In the case of Xpert, we estimated that, in a setting where one in ten individuals tested actually had TB and Xpert could be deployed at high volume, at least 100 four-module machines would be needed to cover a population of 10 million adults. This number would be even higher in a setting where screening of patients with unexplained cough was less efficient (i.e., fewer than one in ten people tested had TB) or Xpert could not be run at high volume (i.e., fewer than 12.5 tests per machine-day). In addition to the logistical hurdles of developing new diagnostic algorithms and increasing laboratory infrastructure, sufficient drug supplies must be made available to handle increased demand (e.g., 15,000 additional MDR-TB diagnoses possible with Xpert over ten years in a city of 10 million adults), and clinic workflow must be changed to prioritize collection of sputum samples early in the day. Although these obstacles are difficult to overcome in many high-burden settings in Africa, innovations in technology and integration of services may make this task more feasible in coming years. Moreover, both same-day microscopy and Xpert scale-up are likely to have other beneficial effects not captured in this analysis. Use of a rapid, accurate test such as Xpert may increase physicians' index of suspicion for TB and their confidence in the laboratory system; implementation of same-day microscopy would likely support infrastructure (e.g., rapid triage of coughing patients, quality assurance protocols, systems for transporting specimens and reporting results, ready drug supplies) that could improve the management of diseases other than TB as well. Despite the difficulty inherent in explicitly modeling these various logistical hurdles, our transparent and generalizable modeling framework provides useful information to decision-makers in the field. For example, by understanding that the idealized impact of optimized smear microscopy is similar to that of replacing smear by Xpert, decision makers can prioritize these options based on the logistical hurdles and resource requirements faced in their setting rather than on expected differences in epidemiological impact.

This analysis has other important limitations. As with other TB modeling analyses [10], [38], [42], we made certain simplifying assumptions (e.g., homogeneous mixing, focus on adult forms of TB, no explicit incorporation of drug-resistant TB, two compartments of HIV, continuous rate of diagnosis and treatment during active TB, linear trajectories of parameters over time) that allowed us to understand the behavior of a complex system and generate reproducible estimates. Although our parsimonious model reduces the number of parameters requiring assumptions, it does not capture complexities (e.g., increasing likelihood of diagnosis with disease progression) that may play an important role in determining the impact of diagnostic interventions on the epidemiology of TB. As shown in Figure 3, certain natural history parameters have a strong influence on our estimates, and the values of these parameters remain poorly understood. Other future externalities – including the trajectory of the HIV epidemic and the availability of other TB diagnostics – cannot be predicted and may also affect the course of the TB epidemic. Our projections should therefore not be taken as realistic estimates of the future course of TB in Africa after scaling-up different diagnostic algorithms, but rather as a tool that uses “best-case scenarios” to assess the comparative population-level impact achievable with idealized implementation of diagnostic strategies. In this regard, our estimates of the comparative effectiveness of same-day microscopy and centralized Xpert are more salient than our absolute estimates of the trajectory of the TB epidemic under each scenario. Furthermore, we provide a transparent model that may be adapted to local settings to provide more locally relevant estimates than the generic projections presented here.

In conclusion, same-day microscopy and Xpert MTB/RIF are complementary diagnostic strategies with the potential to avert substantial morbidity and mortality in communities within the WHO African Region over a 10-year period. The population-level effectiveness of same-day microscopy and Xpert – each implemented in isolation – is similar (9–11% reduction in incidence), but a combined diagnostic strategy including both interventions had additive impact, reducing annual TB mortality by an estimated 44% relative to the current standard of care. Scale-up of Xpert and other novel diagnostics does not ameliorate the need to optimize smear microscopy, nor will same-day microscopy services lessen the potential impact of novel diagnostic tests. Ultimately, a combination of diagnostic improvements – addressing both diagnostic sensitivity and the speed of initiating treatment – and other TB control strategies will be necessary to make transformative progress toward TB elimination.

## Supporting Information

Information S1 **Model Description and Equations.** Full description of mathematical model and symbolic representation of equations.
(DOC)

Information S2 **Model Code.** Model code in R format.
(R) Click here for additional data file.

Information S3 **Equilibrium Population.** Comma-separated value (CSV) file containing the equilibrium population, required for running of the model code (Information S2).
(CSV)
