# Supplementary material for: Population-Level Impact of Same-Day Microscopy and Xpert MTB/RIF for Tuberculosis Diagnosis in Africa
Source: PLoS One. 2013 Aug 12;8(8):e70485. doi: 10.1371/journal.pone.0070485 (PMC3741313; doi:10.1371/journal.pone.0070485)
Supplement: Information S1 — Model Description and Equations. Full description of mathematical model and symbolic representation of equations. (DOC) [file pone.0070485.s001.doc]

**Population-Level Impact Achievable with Same-Day Microscopy and Xpert MTB/RIF for Tuberculosis in Africa**

David W. Dowdy, J. Lucian Davis, Saskia den Boon, Nicholas D. Walter, Achilles Katamba, Adithya Cattamanchi

**Supporting Information S1: Model Description and Equations**

**Model Description**

The mathematical model consists of five TB compartments, each of which allows two HIV states (for a total of ten compartments overall):

- *U_i_*, Uninfected
- *L_i_*, Latently infected
- *A_i_,* Active, smear-positive
- *N_i_,* Active, smear-negative
- *R_i_*, Recovered/Treated

where the subscript *i* denotes two subpopulations: HIV-negative (*i* = 0) and HIV-positive (*i* = 1).

The model assumes an adult population with no immigration or emigration, and with the size of all compartments (i.e., overall population size) increasing at a defined rate. Since, on a scale of 20 years in a model of an adult-only population, this growth reflects more a reduction in mortality than an increase in birth, we assume that this growth occurs proportionally across all compartments. In addition to this constant growth, which occurs across all compartments, deaths are matched by entries into the population. For these entries, individuals enter at age 15 and are assumed to be HIV-uninfected, with no history of active TB, at that time. Thus, these individuals enter the compartments *U_0_* and *L_0_*. Although some studies (e.g., Wood R et al, Int J Tuberc Lung Dis 2010; 14:406) have suggested that the annual risk of TB infection in African settings may increase after the end of adolescence, for simplicity we assume a constant risk of TB infection per year. Thus, the proportion entering the latently-infected compartment *U_0_* is determined at equilibrium (year 2002) as (1 – *e^-15*λ^*), where *λ* is the force of infection as below. For model simplicity (and because the force of infection does not decline rapidly during the ten-year model period), this proportion is held constant throughout the rest of each simulation.

Individuals exit the uninfected compartment *U_i_* at a rate that is determined by the per-person TB transmission rate, number of infectious TB cases (Σ*A_i_* + *σ*Σ*N_i_*), and the relative transmission rate *σ* of each infectious compartment. A proportion of these individuals progress rapidly to active disease *A_i_* or *N_i_*; the remainder progress to the latently infected state (*L_i_*), from which reactivation as well as reinfection and subsequent rapid progression to active disease (at a reduced rate) are both allowed. After progressing to active TB, individuals can enter the recovered compartment *R_i_* through diagnosis and treatment or spontaneous recovery. We assume that diagnosis and treatment occur at a constant annual rate that is a function of an intrinsic probability of undergoing diagnosis per unit time (conditional on HIV status), the sensitivity of the diagnostic test (conditional on smear status), and the probability of treatment success. The probability of undergoing diagnosis may be conceptualized as a constellation of type and severity of symptoms, access to care, patient and health system delays, and type of health facility available; these factors combine to create an intrinsic rate at which diagnosis (leading to effective treatment) is likely to happen. This rate is then multiplied by diagnostic sensitivity and treatment success to create a rate of successful treatment, which is in turn modeled as immediate elimination of infectiousness and return of mortality risk to the baseline state. Unsuccessful treatment is modeled as return to the active TB state from which the patient came. Relapse from *R_i_* to *A_i_* and *N_i_* was considered by fitting to the proportion of notified TB cases that were retreatment, but the best-fitting parameter value was zero, so we removed the parameter from the model. HIV is modeled as a constant incidence rate from *X_0_* to *X_1_*, where *X* represents any of the five TB states listed above; this transition is irreversible. Simultaneous transition of TB and HIV states is not allowed.

**Model Parameters**

The following Table provides all model parameters and their symbolic representations. The corresponding model values are given in Tables 1 and 2 in the main text; those with a subscript *i* have different values for HIV-infected and HIV-uninfected individuals.

| **Parameter** | **Representation** |
| --- | --- |
| Rate of population increase (per year) | *ω* |
| Transmission rate (transmission events per infectious person-year) | *β* |
| Relative risk of TB infection, HIV-positive | *ζ* |
| HIV incidence rate, per year | *η* |
| Rate of diagnostic attempts, per year | *τ_i_* |
| Proportion of recent infections resulting in rapid progression | *π_i_* |
| Reduction in TB rapid progression in people with latent TB infection | *ψ_0_* |
| Reactivation rate after latent infection, per year | *ε_i_* |
| Proportion of TB that is smear-positive | *ρ_i_* |
| Relative infectiousness of smear-negative TB | *σ* |
| Proportion of smear-positive cases missed by sputum microscopy | *δ* |
| Proportion of TB cases lost to follow-up (“initial default”) | *φ(t)* |
| Proportion of TB cases treated empirically | *ξ_e_* |
| Sensitivity of Xpert MTB/RIF for smear-negative TB | *ξ_XP_* |
| Treatment success proportion | *θ* |
| Spontaneous cure rate (smear-positive), per year | *ν_sp_* |
| Spontaneous cure rate (smear-positive), per year | *ν_sn_* |
| Non-TB mortality rate, per year | *μ_i_* |
| TB-specific mortality rate:  HIV-negative, smear-positive  HIV-negative, smear-negative  HIV-positive | *μ_sp0_*  *μ_sn0_*  *μ_sp1_ = μ_sn1_* |

**Model Equations**

Rates of flow between compartments are governed by the system of ordinary differential equations listed in Equations 1-6. We first define the force of infection, total mortality rate, and rates of successful treatment for simplicity. The model was programmed in R version 2.13.1 (R Foundation for Statistical Computing, 2011), and differential equations were solved with the deSolve package at time steps of 0.01 years. The source code for the model is available as Supporting Information S2; this code requires a starting population, provided as Supporting Information S3.

Force of Infection *(λ)*

*λ_i_ = β * ζ_i_ *(Σ_i_A_i_ + σ* Σ_i_N_i_)/ Σ_i_(U_i_ + L_i_ + A_i_ + N_i_ + R_i_)*

TB infection is modeled as a density-dependent process, a function of the transmission rate (*β*), relative risk of TB infection among those who are HIV-infected (*ζ_i_* = 1 if *i* = 0), number of individuals with infectious TB (*A*, smear-positive; *N*, smear-negative) weighted by the relative transmission rate *σ*, and the total size of the population.

Total Mortality Rate *(M)*

*M = Σ_i_[(μ_i_)*(U_i_ + L_i_ + A_i_ + N_i_ + R_i_)] + μ_sp0_*A_0_ + μ_sn0_*N_0_ + μ_sp1_*A_1_ + μ_sn1_*N_1_*

Total mortality is the sum of baseline mortality across all compartments, plus TB-specific mortality across the four compartments that define active TB.

Successful Treatment Rate, Smear-Positive *(χ_spi_)*

*χ_spi_ = τ_i_* θ *{(1 – δ)*(1 – φ)+[1 – (1 – δ)*(1 – φ(t))]* ξ_e_}*

The rate of treatment among smear-positive TB cases is equal to the rate of diagnostic attempts *τ_i_*, scaled by the treatment success proportion *θ*, the sensitivity of sputum smear for smear-positive disease *(1 – δ)*, and one minus the “initial default” proportion *(1 – φ(t))*. (The “initial default” proportion is stable at 0.15 in the baseline scenario but declines linearly to 0.015 over 1.8 years in the same-day microscopy and same-day Xpert scenarios.) Among those who are either false-negative or initial defaulters, a proportion *ξ_e_* are treated empirically (e.g., symptoms so classic or so severe that physicians are willing to treat without smear results).

Successful Treatment Rate, Smear-Negative *(χ_sni_)*

*χ_sni_ = τ_i_* θ *{ξ_e_ +(1 – ξ_e_)* [ξ_XP_*(1 – φ(t))*(t/2)] }*

The rate of treatment among smear-negative TB cases is equal to the the rate of diagnostic attempts *τ_i_*, scaled by the treatment success proportion *θ* and the proportion proportion *ξ_e_* treated empirically. Among those who would not be treated empirically *(1 – ξ_e_)*, Xpert MTB/RIF diagnoses a proportion of individuals *ξ_XP_*, subject to “initial default” *(1 – φ(t))*. As Xpert ss assumed to be scaled-up over time, the proportion of diagnoses made by Xpert increases over time *t* (measured in years, with time 0 being January 1, 2013).

*Equation 1. Uninfected Compartment (U_i_)*

*dU_i_/dt = ω*U_i_ + [(1 – i)*(e^-15*λ0^)*M] – (λ_i_ + μ_i_) * U_i_ + yηU_0_*

where *ω* is the constant rate of population increase, *(1 – i)* denotes that entries (to match deaths) are all HIV-uninfected, *(e^-15*λ0^)* is the equilibrium proportion of 15-year-olds who are uninfected, *M* is the total mortality as defined above (matched to provide a constant population except for *ω*), *λ_i_* is the force of infection, *μ_i_* is the non-TB mortality rate, and *y* [= -1 if HIV-negative, +1 if HIV-positive]*η* is the HIV infection rate. Thus, uninfected individuals leave this compartment through infection and death, move from *U_0_* to *U_1_* through HIV infection, and *U_0_* is replenished at a rate that, when combined with additions to *L_0_*below, matches total mortality.

*Equation 2. Latently Infected Compartment (L_i_)*

*dL_i_/dt = ω*L_i_ +[(1 – i)*(1 – e^-15*λ0^)*M] + [λ_i_ * (1 – π_i_)] * U_i_*

*– [λ_i_ * π_i_ * (1 – ψ_i_) + ε_i_ + μ_i_] * L_i_ + yηL_0_*

where *ω* is the constant rate of population increase*,* *(1 – i)* denotes that entries (to match deaths) are all HIV-uninfected, *(1 – e^-15*λ0^)* is the equilibrium proportion of 15-year-olds who are latently infected, *M* is the total mortality as defined above (matched to provide a constant population except for *ω*), *λ_i_* is the force of infection, *π_i_* is the proportion of recent infections that progress rapidly to active TB, *ψ_i_* is the relative reduction in rapid progression after infection among people with latent TB, *ε_i_* is the endogenous reactivation rate, *μ_i_* is the non-TB mortality rate, and *y* [= -1 if HIV-negative, +1 if HIV-positive]*η* is the HIV infection rate. Thus, susceptible individuals who do not progress rapidly are modeled as developing latent infection, and latently-infected individuals leave this compartment through TB reinfection (followed by rapid progression), endogenous reactivation, and death.

*Equation 3. Active, Smear-Positive Compartment (A_i_)*

*dA_i_/dt = ω*A_i_ + ρ_i_ *{(λ_i_ * π_i_) * [U_i_ + (1 – ψ_i_) * ( L_i_ + R_i_)]}*

*+ ρ_i_ *[ε_i_ * (L_i_ + R_i_)]*

*– (χ_spi_ + μ_i_ + μ_spi_ + ν_spi_) * A_i_**+ yηL_0_*

where *ω* is the constant rate of population increase*,* *ρ_i_* is the proportion of TB that is smear-positive, *λ_i_* is the force of infection, *π_i_* is the proportion of recent infections that progress rapidly to active TB, *ψ_i_* is the relative reduction in rapid progression after infection among people with latent TB, *ε_i_* is the endogenous reactivation rate, *χ_spi_* is the successful treatment rate for smear-positive TB, *μ_i_* is the non-TB mortality rate, *μ_spi_* is the smear-positive TB mortality rate, *ν_spi_* is the spontaneous cure rate of smear-positive TB, and *y* [= -1 if HIV-negative, +1 if HIV-positive]*η* is the HIV infection rate. Thus, individuals enter this compartment through rapid progression of recent infection and endogenous reactivation of latent infection (including those who have recovered from prior disease). They exit through successful treatment, spontaneous cure, and death.

*Equation 4. Active, Smear-Negative Compartment (A_i_)*

*dN_i_/dt = ω*N_i_ + (1 – ρ_i_) *{(λ_i_ * π_i_) * [U_i_ + (1 – ψ_i_) * ( L_i_ + R_i_)]}*

*+(1 – ρ_i_) *[ε_i_ * (L_i_ + R_i_)]*

*– (χ_sni_ + μ_i_ + μ_sni_ + ν_sni_) * N_i_**+ yηL_0_*

where *ω* is the constant rate of population increase*,* *(1 – ρ_i_)*  is the proportion of TB that is smear-negative, *λ_i_* is the force of infection, *π_i_* is the proportion of recent infections that progress rapidly to active TB, *ψ_i_* is the relative reduction in rapid progression after infection among people with latent TB, *ε_i_* is the endogenous reactivation rate, *χ_sni_* is the successful treatment rate for smear-negative TB, *μ_i_* is the non-TB mortality rate, *μ_sni_* is the smear-negative TB mortality rate, *ν_sni_* is the spontaneous cure rate of smear-negative TB, and *y* [= -1 if HIV-negative, +1 if HIV-positive]*η* is the HIV infection rate. Thus, individuals enter this compartment through rapid progression of recent infection and reactivation of latent infection (including those who have recovered from prior disease). They exit through treatment, spontaneous cure, and death.

*Equation 5. Recovered/Treated Compartment (R)*

*dR/dt = ω*R_i_+ (χ_spi_ + ν_spi_) * A_i_ + (χ_sni_ + ν_sni_) * N_i_ – [λ_i_ * π_i_ * (1 – ψ_i_) + ε_i_ + μ_i_]* R_i_*

where *ω* is the constant rate of population increase*,* *χ_spi_* and *χ_sni_* are the successful treatment rates for smear-positive and smear-negative TB, *ν_spi_* and *ν_sni_* are the spontaneous cure rates of smear-positive and smear-negative TB *μ_i_* is the non-TB mortality rate, , *λ_i_* is the force of infection, *π_i_* is the proportion of recent infections that progress rapidly to active TB, *ψ_i_* is the relative reduction in rapid progression after infection among people with latent TB (including those who have recovered), *ε_i_* is the endogenous reactivation rate, and *μ_i_* is the non-TB mortality rate. Thus, all individuals who are treated or spontaneously recover enter this compartment, and individuals leave the compartment through TB reinfection (followed by rapid progression), endogenous reactivation, and death.
